# Clinicopathologic characteristics and survival outcomes of gastroenteropancreatic neuroendocrine neoplasms in Peru: A 15-year retrospective cohort study

**DOI:** 10.1371/journal.pone.0361867

**Published:** 2026-11-05

**Authors:** Wagner Eduardo Cruz-Diaz, Juan Haro-Varas, Daniel Enriquez-Vera, Gabriel De la Cruz Ku, Nattaly Torres-Valdivia, Víctor Paitán Amaro, Alexandra Saavedra, Alejandro Merubia, Angela Leonardo Carbajal, Jackeline Macetas Núñez, Eder Veramendi Cabana, Cristian Pacheco Román, Mónica Calderón Anticona, Tatiana Vidaurre Rojas, Victor Castro-Oliden

**Affiliations:** 1 Instituto Nacional de Enfermedades Neoplásicas, Lima, Peru; 2 Escuela de Medicina Humana, Universidad Privada San Juan Bautista, Lima, Peru; 3 Universidad Científica del Sur, Lima, Peru; Huazhong Agriculture University, CHINA

## Abstract

**Introduction:**

Incidence of gastroenteropancreatic neuroendocrine neoplasms (GEP-NENs) has increased globally; however, data from Latin America remain scarce. This study aimed to describe the sociodemographic and clinicopathologic characteristics, treatment modalities, survival outcomes, and prognostic factors of GEP-NENs in a 15-year single-center cohort at a national referral cancer center in Lima, Peru.

**Materials and methods:**

We conducted a retrospective, hospital-based cohort study. Patients diagnosed with GEP-NENs at the Instituto Nacional de Enfermedades Neoplásicas (INEN) were included from 2009 to 2024, and tumors were classified according to the 2019 WHO Classification of GEP-NENs. Overall survival (OS) was estimated using Kaplan-Meier curves and compared using the log-rank test. Prognostic factors were identified through Cox proportional hazards models.

**Results:**

Three hundred sixty-eight patients were included. The median age at diagnosis was 57 years, with a female predominance (56.2%). Most patients presented with an ECOG performance status of 0–1 (96.5%), and among patients with available disease-extent information, 64.7% had non-metastatic disease. The majority of tumors were well-differentiated (neuroendocrine tumors [NETs]) (84.2%), while 15.8% were poorly differentiated (neuroendocrine carcinomas [NECs]). The most common primary tumor sites included the rectum (39.1%), stomach (19.8%), and pancreas (18.8%). With a median follow-up of 61.9 months, the 5-year OS rate was 52%. NETs exhibited longer OS than NECs, with 5-year OS rates of 65% and 8%, respectively (log-rank p < 0.001). In the multivariable analysis, metastatic disease (aHR = 2.61, 95% CI: 1.63–4.20; p < 0.001) and poorly differentiated histology (aHR: 4.36, 95% CI: 2.59–7.31; p < 0.001) were independently associated with worse overall survival, whereas surgery (aHR: 0.25, 95% CI: 0.15–0.41; p < 0.001) and systemic treatment (aHR: 0.53, 95% CI: 0.31–0.92; p = 0.024) were associated with improved overall survival.

**Conclusion:**

Our findings demonstrate that GEP-NENs represent a highly heterogeneous group of neoplasms with substantial variability in clinicopathological characteristics and survival outcomes. Most patients in our cohort had well-differentiated NETs and favorable long-term survival. These findings support the need for comprehensive national registries and multicenter collaborative studies to validate our results and better characterize GEP-NENs in underrepresented Latin American populations.

## Introduction

Neuroendocrine neoplasms (NENs) constitute a heterogeneous group of malignancies arising from neuroendocrine cells distributed throughout the body. Approximately two-thirds of these neoplasms originate from the gastroenteropancreatic (GEP) system [1]. Historically, GEP-NENs were considered rare tumors; however, in recent decades, their incidence has increased globally [1–8].

The incidence of GEP-NENs varies across racial and ethnic groups [9]. Data from the U.S. Surveillance, Epidemiology, and End Results (SEER) 17 indicate an incidence of 4.27 per 100,000 persons for GEP-NENs, with the highest rate in small intestine NENs (siNENs) [2]. In contrast, population-based data from China reported an age-standardized incidence rate of NENs of 1.14 per 100,000 persons in 2017, with pancreatic NENs (pNENs) as the most frequent primary site [3].

Epidemiological data on GEP-NENs in Latin America (LATAM) remain limited. Most available evidence derives from populations with predominant European ancestry, whereas countries with a high proportion of Native American ancestry—such as Peru, Bolivia, and Ecuador—are markedly underrepresented, limiting insight into regional heterogeneity within Hispanic/Latino populations. An observational study conducted in Argentina documented 532 NENs, of which 461 were GEP-NENs. The most common were siNENs (26.9%) and pNENs (25.2%) [10]. A NEN registry from Brazil collected baseline data on the first 1,000 patients enrolled across 32 centers throughout all regions of the country. It identified GEP-NENs as the second most common type, accounting for 20.2% of cases [11]. In Peru, a retrospective review at INEN identified 367 patients diagnosed with NENs from 2010 to 2014, with GEP-NENs accounting for 44.8% of cases [12].

Consequently, we aimed to describe the sociodemographic and clinicopathologic characteristics, treatment modalities, survival outcomes, and prognostic factors of GEP-NENs over a 15-year period at a national referral cancer center in Lima, Peru.

## Materials and methods

### Study design and setting

We conducted a retrospective, hospital-based cohort study of patients diagnosed with GEP-NENs and treated at the “Instituto Nacional de Enfermedades Neoplásicas” in Lima, Peru, between January 2009 and December 2024. The patients were followed until December 2025. Medical records were reviewed, and data were collected from July 2025 to January 2026. Our inclusion criteria encompassed patients aged 18 years or older with a confirmed diagnosis of GEP-NENs, with disease classified as either non-metastatic or metastatic at the time of diagnosis. Additionally, all specimens were confirmed by the institute’s Pathology Department, with each case reviewed by at least two pathologists. We excluded patients with incomplete pathological data, including Ki-67 assessment, and those who were lost to follow-up were censored at the date of last contact. No patients involved in this study were part of national or international clinical trials.

### Variables

Sociodemographic and clinicopathological characteristics were collected. Tumors were classified according to the 2019 WHO Classification of Tumours of the Digestive System [13]. Tumor differentiation was classified as well-differentiated or poorly differentiated based on histopathological assessment of tumor morphology. Tumor grade was assigned according to the Ki-67 proliferation index using the following thresholds: G1 (<3%), G2 (3–20%), and G3 (>20%). According to the 2019 WHO Classification of Tumours of the Digestive System, well-differentiated NET G3 and poorly differentiated NEC were considered distinct entities. Accordingly, all poorly differentiated G3 neoplasms were classified as NECs, whereas well-differentiated neoplasms with a Ki-67 index >20% were classified as NET G3. Detailed AJCC 8th edition TNM clinical staging was not consistently available throughout the 15-year study period. Therefore, disease extent at diagnosis was harmonized into two categories for descriptive and survival analyses: non-metastatic disease, defined as the absence of documented distant metastasis and encompassing both localized and regional disease, corresponding to AJCC 8th edition stages I-III; and metastatic disease, defined as the presence of documented distant metastasis, corresponding to AJCC 8th edition stage IV [14]. Regional and metastatic disease were therefore not grouped together. The primary tumor site was confirmed by imaging studies and biopsy. Treatment modalities were categorized as surgery, systemic therapy, radiotherapy, palliative, or combined therapy. Treatment categories reflected the principal treatment modality documented during the patient’s management at our institution rather than treatment received at a predefined time point or specific line of therapy. Surgery included curative-intent resection for patients with non-metastatic disease and, when documented in the medical record, selected cytoreductive or palliative procedures for patients with metastatic disease. Systemic therapy included somatostatin analogs or chemotherapy. Missing treatment information was not considered equivalent to absence of treatment and was excluded from analyses requiring the corresponding treatment variable. For systemic therapy regimen, patients without documented systemic therapy were considered not applicable. Because treatment intent, surgical radicality, sequence of systemic therapy, cytoreductive thresholds, and subsequent treatment lines were not uniformly documented throughout the study period, treatment-related survival analyses were considered exploratory and should be interpreted with caution. Overall survival (OS) was defined as the time from primary tumor diagnosis to death or study completion. All patients were treated in accordance with institutional protocols based on National Comprehensive Cancer Network guidelines.

### Statistical analysis

Descriptive statistics were used to assess sociodemographic, clinical, pathological, surgical, and therapeutic characteristics. We used median with interquartile range (IQR) for quantitative variables; frequencies and percentages were used to assess qualitative variables. Stratified analysis was performed according to age, tumor differentiation, and primary site. Kaplan-Meier method was used to estimate survival probabilities, while the log-rank test was used to assess the differences between categories. Survival outcomes were evaluated at a 5-year follow-up period. Patients who were lost to follow-up were censored at the date of last contact. Missing data were reported in the tables and not included in our analyses. Prognostic factors were evaluated using multivariable Cox proportional hazards regression analysis, and hazard ratios (HRs) with 95% confidence intervals (CIs) were reported. Because tumor grade and tumor differentiation are closely related pathological characteristics, sensitivity analyses were performed using two separate multivariable Cox proportional hazards models in which these variables were evaluated independently. Model A included tumor differentiation and excluded tumor grade, whereas Model B included tumor grade and excluded tumor differentiation. The proportional hazards assumption was evaluated using Schoenfeld residuals for the primary multivariable model and both sensitivity models. No significant global violations of the proportional hazards assumption were identified (primary model, p = 0.38; Model A, p = 0.45; Model B, p = 0.33). In addition, stage-stratified Kaplan-Meier survival analyses and multivariable Cox proportional hazards regression models were performed separately for patients with non-metastatic and metastatic disease to further evaluate the association between treatment and overall survival. Because this was a retrospective cohort study, all eligible patients meeting the inclusion criteria during the study period were included, and no a priori sample size calculation was performed. The adequacy of the multivariable Cox regression model was assessed according to the number of observed outcome events relative to the number of estimated regression parameters (events-per-variable approach). A 95% CI was used, and a p-value <0.05 was considered statistically significant. All data were analyzed following standard procedures with R and RStudio using the packages tidyverse, ggplot2, sandwich, finalfit, and survminer.

### Ethics

This study was approved by the Institutional Review Board (IRB) from the “Instituto Nacional de Enfermedades Neoplásicas” (INEN 25−18). Due to the retrospective design of our study, consent was waived by the ethics committee. Data were collected confidentially by the investigators after obtaining authorization to access the medical records by the IRB at the institution. All data were handled with strict confidentiality and used only for study purposes; the data were de-identified, and clinical information was coded. The deidentified study dataset is provided in S1 File.

## Results

### Baseline characteristics

A total of 1,090 patients with a diagnosis of NENs were identified from institutional databases from 2009–2024. After removal of duplicate records (n = 241), 849 unique patients remained. Non-gastroenteropancreatic NENs (n = 408) were subsequently excluded, leaving 441 patients with GEP-NENs. Of these, 73 patients were excluded because pathology specimens were unavailable for institutional review. The final study population therefore comprised 368 patients with GEP-NENs (Fig 1). The median age at diagnosis was 57 years (IQR: 47–65 years), with a female predominance of 56.2%. The majority of patients (96.5%) presented with good performance status (ECOG 0–1). Among patients with available disease-extent information (n = 334), 216 (64.7%) had non-metastatic disease and 118 (35.3%) had metastatic disease; disease extent was unavailable for 34 patients. The most common primary sites were the rectum (39.1%), stomach (19.8%), and pancreas (18.8%).

**Fig 1:**
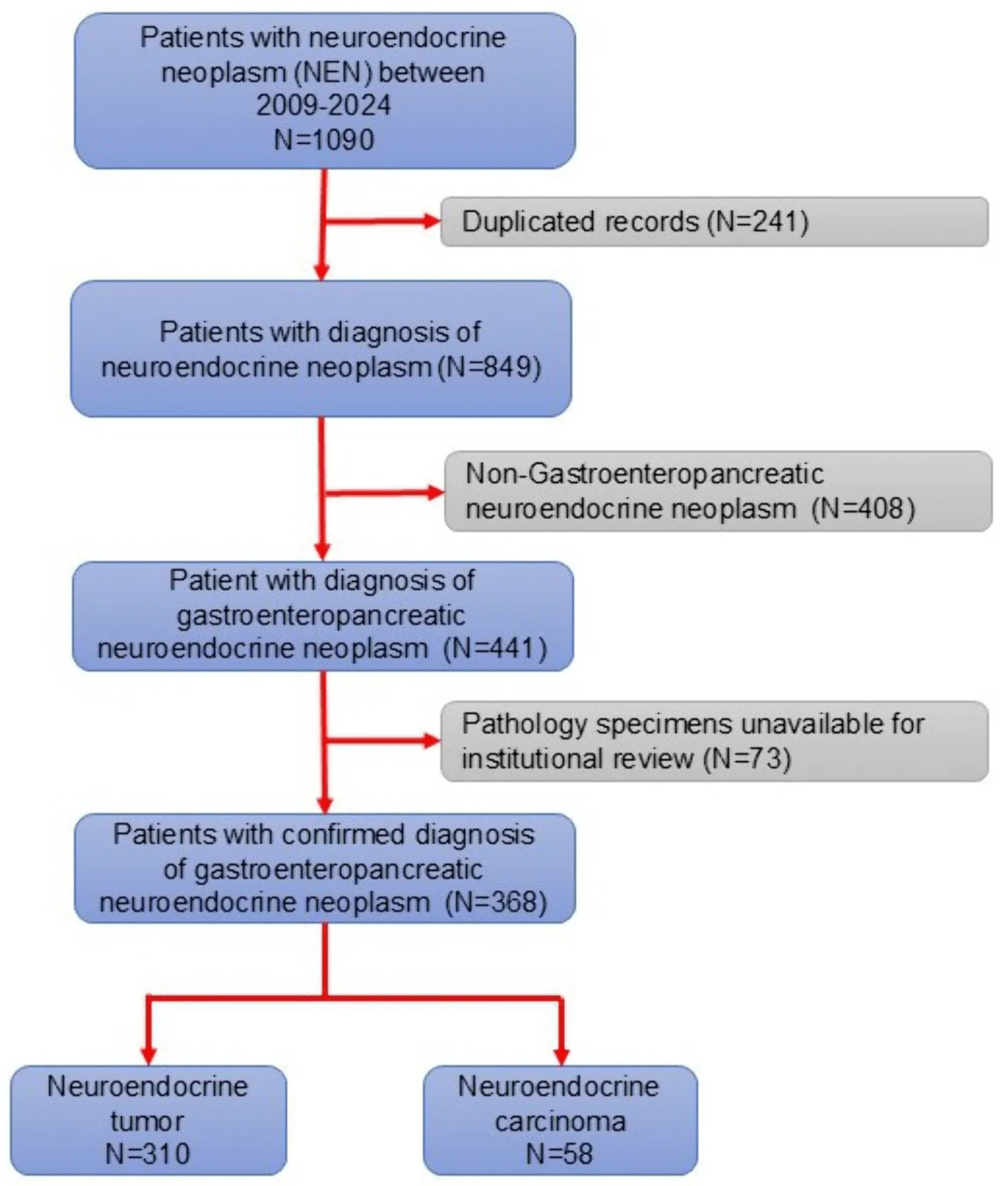
Flow diagram of patient inclusion in the study.

Histologically, 84.2% were well-differentiated neuroendocrine tumors (NETs) and 15.8% were poorly differentiated neuroendocrine carcinomas (NECs). Overall baseline characteristics and differences between NETs and NECs are presented in Table 1. Patients with NETs were significantly younger than those with NECs (56 years vs. 63 years; p < 0.001). Performance status was more preserved in NETs than NECs (ECOG 0–1: 98.1% vs. 88.2%; p = 0.004). NETs more frequently presented with non-metastatic disease than NECs (69.6% vs. 37.3%; p < 0.001). Primary tumor site distribution differed significantly between NETs and NECs (p < 0.001). Rectal tumors were more frequent among NETs (43.2%), whereas gallbladder tumors occurred exclusively among NECs (n = 11); all appendiceal tumors were NETs (n = 8). As expected, the Ki-67 index was significantly lower in NETs compared to NECs (2% vs. 65%; p < 0.001).

**Table 1:** Baseline demographic and clinicopathologic characteristics of a Peruvian cohort of gastroenteropancreatic neuroendocrine neoplasms, stratified by tumor differentiation.

Variable	Total N = 368	NET, N = 310 (84.2%)	NEC, N = 58 (15.8%)	p-value
**Median age/years (IQR)**	57 (47, 65)	56 (45, 64)	63 (55, 72)	<0.001
**Age groups**				<0.001
<65	269 (73.1)	238 (76.8)	31 (53.4)	
≥65	99 (26.9)	72 (23.2)	27 (46.6)	
**Sex**				0.2
Male	161 (43.8)	131 (42.3)	30 (51.7)	
Female	207 (56.2)	179 (57.7)	28 (48.3)	
**Region of origin**				0.5
Amazonian region	15 (4.8)	14 (5.5)	1 (1.8)	
Andean region	96 (30.7)	76 (29.7)	20 (35.1)	
Coastal region	202 (64.5)	166 (64.8)	36 (63.2)	
Unknown	55	54	1	
**ECOG**				0.004
≤1	300 (96.5)	255 (98.1)	45 (88.2)	
2	11 (3.5)	5 (1.9)	6 (11.8)	
Unknown	57	50	7	
**Tumor primary site**				<0.001
Appendix	8 (2.2)	8 (2.6)	0 (0)	
Colon	21 (5.7)	15 (4.8)	6 (10.3)	
Esophagus	1 (0.3)	0 (0)	1 (1.7)	
Gallbladder	11 (3.0)	0 (0)	11 (19)	
Pancreas	69 (18.8)	57 (18.4)	12 (20.7)	
Rectum	144 (39.1)	134 (43.2)	10 (17.2)	
Small bowel	41 (11.1)	38 (12.3)	3 (5.2)	
Stomach	73 (19.8)	58 (18.7)	15 (25.9)	
**Disease extent at diagnosis**				<0.001
Metastatic	118 (35.3)	86 (30.4)	32 (62.7)	
Non-metastatic	216 (64.7)	197 (69.6)	19 (37.3)	
Unknown	34	27	7	
**Tumor grade**				<0.001
G1	205 (55.7)	205 (66.1)	0 (0)	
G2	102 (27.7)	101 (32.6)	1 (1.7)	
G3	61 (16.6)	4 (1.3)	57 (98.3)	
**Ki-67 index**	2 (1, 10)	2 (1, 5)	65 (40, 90)	<0.001

Data are presented as n (%) unless otherwise indicated. Percentages were calculated using available data for each variable; missing observations were excluded from percentage calculations and are reported separately.

Abbreviations: ECOG, Eastern Cooperative Oncology Group; IQR, interquartile range; NEC, neuroendocrine carcinoma; NET, neuroendocrine tumor.

### Treatment and Survival

Among patients with available treatment information, surgery was the most frequently documented treatment modality in both disease-extent groups and was more common among patients with non-metastatic disease (143/163, 87.7%) than among those with metastatic disease (47/116, 40.5%) (S2 File, Supplementary Table 1). Among patients with available treatment information, systemic therapy included somatostatin analogs and chemotherapy. Among patients with documented systemic therapy regimen information, CAPTEM was the most frequently documented regimen for NETs, whereas platinum-etoposide was the predominant regimen for NECs. Only 5 cases (1.4%) received combined therapy (Table 2).

**Table 2:** Treatment modalities in a Peruvian cohort of gastroenteropancreatic neuroendocrine neoplasms, stratified by tumor differentiation.

Variable	Total N = 368	NET, N = 310 (84.2%)	NEC, N = 58 (15.8%)	p-value^*a*^
**Treatment modality**				0.002
Combined	5 (1.8)	4 (1.8)	1 (2.0)	
Palliative	38 (13.6)	23 (10)	15 (30.0)	
Radiotherapy	7 (2.5)	5 (2.2)	2 (4.0)	
Surgery	191 (68.2)	168 (73.0)	23 (46.0)	
Systemic	39 (13.9)	30 (13.0)	9 (18.0)	
Missing	88	80	8	
**Systemic therapy regimen among patients with documented regimen information**				<0.001
CAPTEM	19 (55.9)	19 (73.1)	0 (0.0)	
Doxorubicin	1 (2.9)	1 (3.8)	0 (0.0)	
Octreotide	3 (8.8)	3 (11.5)	0 (0.0)	
Platinum-Etoposide	11 (32.4)	3 (11.5)	8 (100.0)	
Missing regimen	5	4	1	

Data are presented as n (%). Percentages were calculated among patients with available treatment information; missing observations were excluded from percentage calculations and are reported separately. Missing treatment information does not indicate absence of treatment.

For systemic therapy regimen, patients without documented systemic therapy were considered not applicable; missing regimen data refer only to patients with documented systemic therapy for whom the specific regimen could not be determined.

Abbreviations: CAPTEM, capecitabine-temozolamide; NEC, neuroendocrine carcinoma; NET, neuroendocrine tumor.

^a^Fisher’s exact test for count data with simulated p-value (based on 2,000 replicates); missing observations were excluded from hypothesis testing.

With a median follow-up of 61.9 months (IQR: 16.5–148.6 months), we observed 85 deaths (27.4%) among patients with NETs and 55 deaths (94.8%) among patients with NECs. The median OS for the entire cohort was 62 months (95% confidence interval [CI]: 42–77 months), with a 5-year OS rate of 52%. NETs demonstrated superior OS, with a 5-year OS of 65%, whereas NECs had a 5-year OS of 8% (log-rank p < 0.001). Survival outcomes varied significantly by tumor primary site (Fig 2). Patients with aNENs (5-year OS: 83%) and siNENs (5-year OS: 64%) achieved the longest survival, whereas those with gbNENs experienced the worst survival (5-year OS: 18%). Survival estimates for primary-site subgroups with very small sample sizes should be interpreted cautiously, particularly for appendiceal (n = 8; 2 deaths), gallbladder (n = 11; 11 deaths), and esophageal NENs (n = 1; 1 death). Table 3 presents the median and 5-year OS rates stratified by patient and tumor characteristics (Figs 3–5). Univariate and multivariable Cox proportional hazards regression analyses are presented in Table 4. In univariate analysis, age ≥ 65 years, male sex, ECOG performance status of 2, gallbladder primary site, metastatic disease at diagnosis, tumor grade G2–G3, and NEC histology were associated with worse OS, whereas combined therapy/radiotherapy and surgery were associated with improved survival. After multivariable adjustment, metastatic disease at diagnosis (adjusted hazard ratio [aHR] = 2.61, 95% CI: 1.63–4.20; p < 0.001), NEC histology (aHR: 4.36, 95% CI: 2.59–7.31; p < 0.001), and tumor grade 2–3 (aHR: 1.86, 95% CI: 1.07–3.23; p = 0.027) remained associated with worse OS, whereas surgery (aHR: 0.25, 95% CI: 0.15–0.41; p < 0.001) and systemic treatment (aHR: 0.53, 95% CI: 0.31–0.92; p = 0.024) were independently associated with improved OS.

**Table 3:** Overall survival according to patient, tumor, and treatment characteristics in a Peruvian referral cohort of gastroenteropancreatic neuroendocrine neoplasms.

Variable	5-year OS (95% CI)	Median OS (95% CI)	p-value
**Overall**	52 (46-60)	62 (42-77)	
**Age groups**			
<65	59 (52-68)	76 (63-97)	<0.001
≥65	34 (24-49)	29 (23-55)	<0.001
**Sex**			
Female	60 (52-70)	77 (63-150)	0.001
Male	42 (33-54)	41 (28-70)	0.001
**ECOG**			
≤1	56 (49-64)	70 (51-90)	<0.001
2	0 (NA-NA)	5 (3-NR)	<0.001
**Primary tumor site**			
Appendix	83 (58-100)	85 (85-NR)	0.006
Colon	36 (17-75)	36 (11-NR)	0.006
Gallbladder	18 (5-64)	3 (3-NR)	0.006
Pancreas	62 (49-80)	78 (51-NR)	0.006
Rectum	47 (36-60)	49 (36-77)	0.006
Small bowel	64 (47-86)	75 (55-NR)	0.006
Stomach/esophagus	57 (44-73)	150 (31-NR)	0.006
**Disease extent at diagnosis**			
Metastatic	32 (24-44)	27 (22-42)	<0.001
Non-metastatic	70 (62-80)	150 (90-NR)	<0.001
**Tumor differentiation**			
NEC	8 (3-20)	10 (5-16)	<0.001
NET	65 (58-74)	85 (74-94)	<0.001
**Treatment modality**			
Combined	0 (NA-NA)	23 (12-NR)	<0.001
Palliative	21 (11-40)	6 (4-28)	<0.001
Radiotherapy	71 (45-100)	NR (2-NR)	<0.001
Surgery	71 (63-80)	97 (90-NR)	<0.001
Systemic	28 (15-52)	34 (13-63)	<0.001
**Tumor grade**			
G1	79 (71-88)	149 (90-NR)	<0.001
G2-G3	32 (24-42)	28 (21-38)	<0.001

Abbreviations: CI, confidence interval; ECOG, Eastern Cooperative Oncology Group; NA, not available; NEC, neuroendocrine carcinoma; NET, neuroendocrine tumor; NR, not reached; OS, overall survival.

^a^P-values were calculated using the log-rank test and represent the overall comparison across categories of each variable.

**Table 4:** Univariate and multivariate Cox proportional hazards analysis of factors associated with overall survival in a Peruvian cohort of gastroenteropancreatic neuroendocrine neoplasms.

Variable	All	HR (95% CI)	p-value	aHR (95% CI)	p-value
**Age group**					
<65	269 (73.1)	1.00	–	1.00	–
≥65	99 (26.9)	2.01 (1.43-2.84)	<0.001	1.46 (0.97-2.19)	0.071
**Sex**					
Female	207 (56.2)	1.00	–	1.00	–
Male	161 (43.8)	1.73 (1.24-2.42)	<0.001	0.66 (0.44-1.02)	0.051
**ECOG**					
≤1	300 (96.5)	1.00	–	1.00	–
2	11 (3.5)	7.45 (3.61-15.34)	<0.001	2.02 (0.91-3.51)	0.086
**Primary tumor site**					
Pancreas	69 (18.8)	1.00	–	1.00	–
Lower gastrointestinal tract	173 (47.0)	0.94 (0.56-1.73)	0.944	1.80 (0.85-3.51)	0.112
Gallbladder	11 (3.0)	3.42 (1.67-6.99)	0.001	1.42 (0.61-3.43)	0.684
Small bowel	41 (11.1)	0.98 (0.52-1.85)	0.943	1.54 (0.54-3.41)	0.452
Stomach/esophagus	74 (20.1)	1.00 (0.56-1.75)	0.988	1.75 (0.91-3.75)	0.112
**Disease extent at diagnosis**					
Non-metastatic	216 (64.7)	1.00	–	1.00	–
Metastatic	118 (35.3)	3.54 (2.44-5.12)	<0.001	2.61 (1.63-4.20)	<0.001
**Tumor differentiation**					
NET	310 (84.2)	1.00	–	1.00	–
NEC	58 (15.8)	5.53 (3.89-7.86)	<0.001	4.36 (2.59-7.31)	<0.001
**Treatment modalities**					
Palliative	38 (13.6)	1.00	–	1.00	–
Combined and Radiotherapy	12 (4.3)	0.38 (0.16-0.91)	0.003	0.60 (0.24-1.51)	0.281
Surgery	191 (68.2)	0.14 (0.09-0.22)	<0.001	0.25 (0.15-0.41)	<0.001
Systemic	39 (13.9)	0.61 (0.37-1.02)	0.058	0.53 (0.31-0.92)	0.024
**Tumor grade**					
G1	205 (55.7)	1.00	–	1.00	–
G2-3	163 (44.3)	3.86 (2.62-5.68)	<0.001	1.86 (1.07-3.23)	0.027

Percentages were calculated using available data for each variable; missing observations were excluded from the corresponding analyses.

Abbreviations: CI, confidence interval; ECOG, Eastern Cooperative Oncology Group; HR, hazard ratio; aHR, adjusted hazard ratio; NEC, neuroendocrine carcinoma; NET, neuroendocrine tumor; NR, not reached; OS, overall survival.

**Fig 2:**
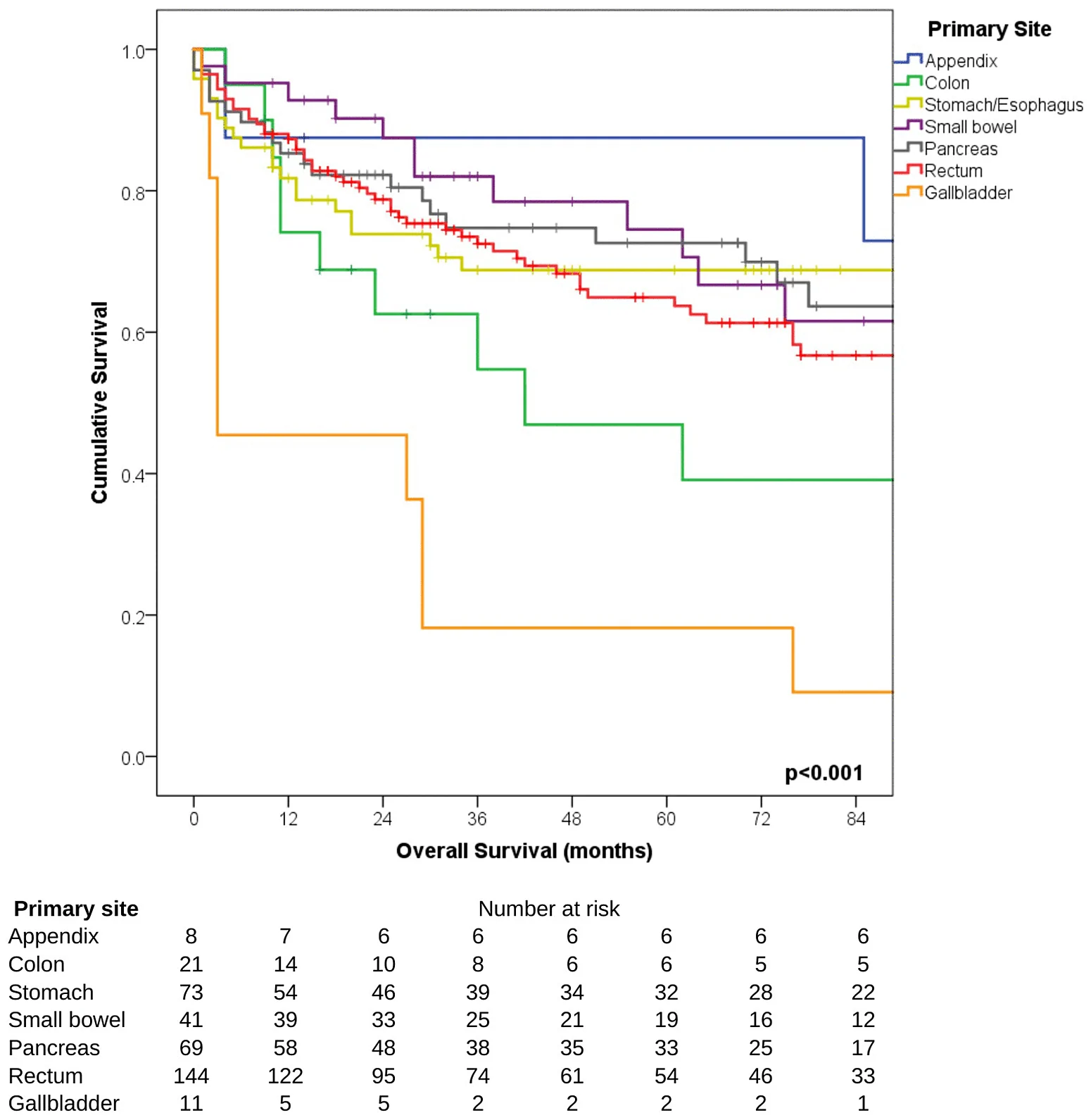
Kaplan-Meier curves showing overall survival according to primary tumor site. Vertical marks indicate censored observations, and numbers at risk are shown below the plot. Corresponding 5-year overall survival estimates with 95% confidence intervals are reported in Table 3. Estimates for primary-site subgroups with small sample sizes should be interpreted cautiously.

**Fig 3:**
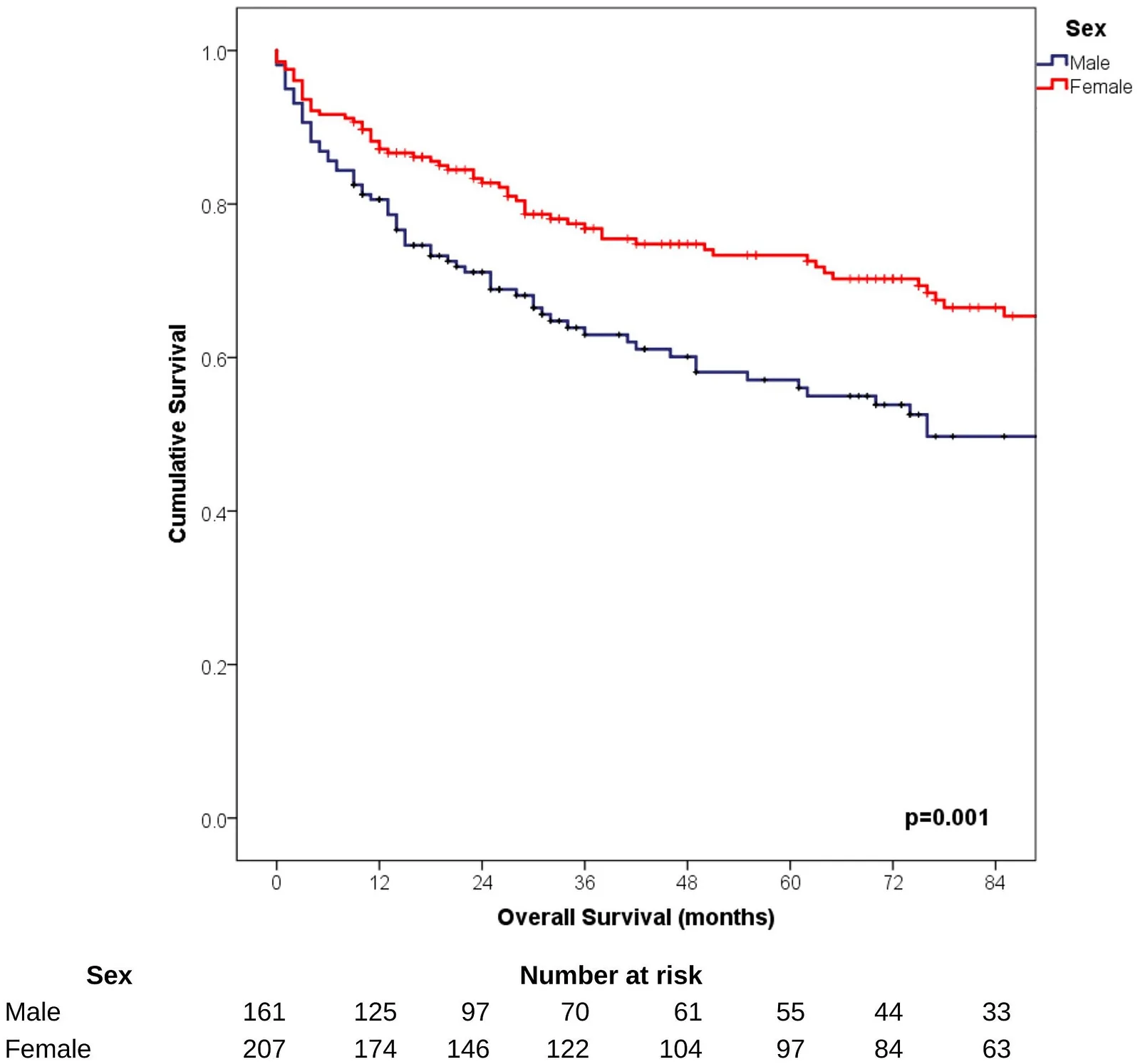
Kaplan-Meier curves showing overall survival stratified by sex. Vertical marks indicate censored observations, and numbers at risk are shown below the plot. Corresponding 5-year overall survival estimates with 95% confidence intervals are reported in Table 3.

**Fig 4:**
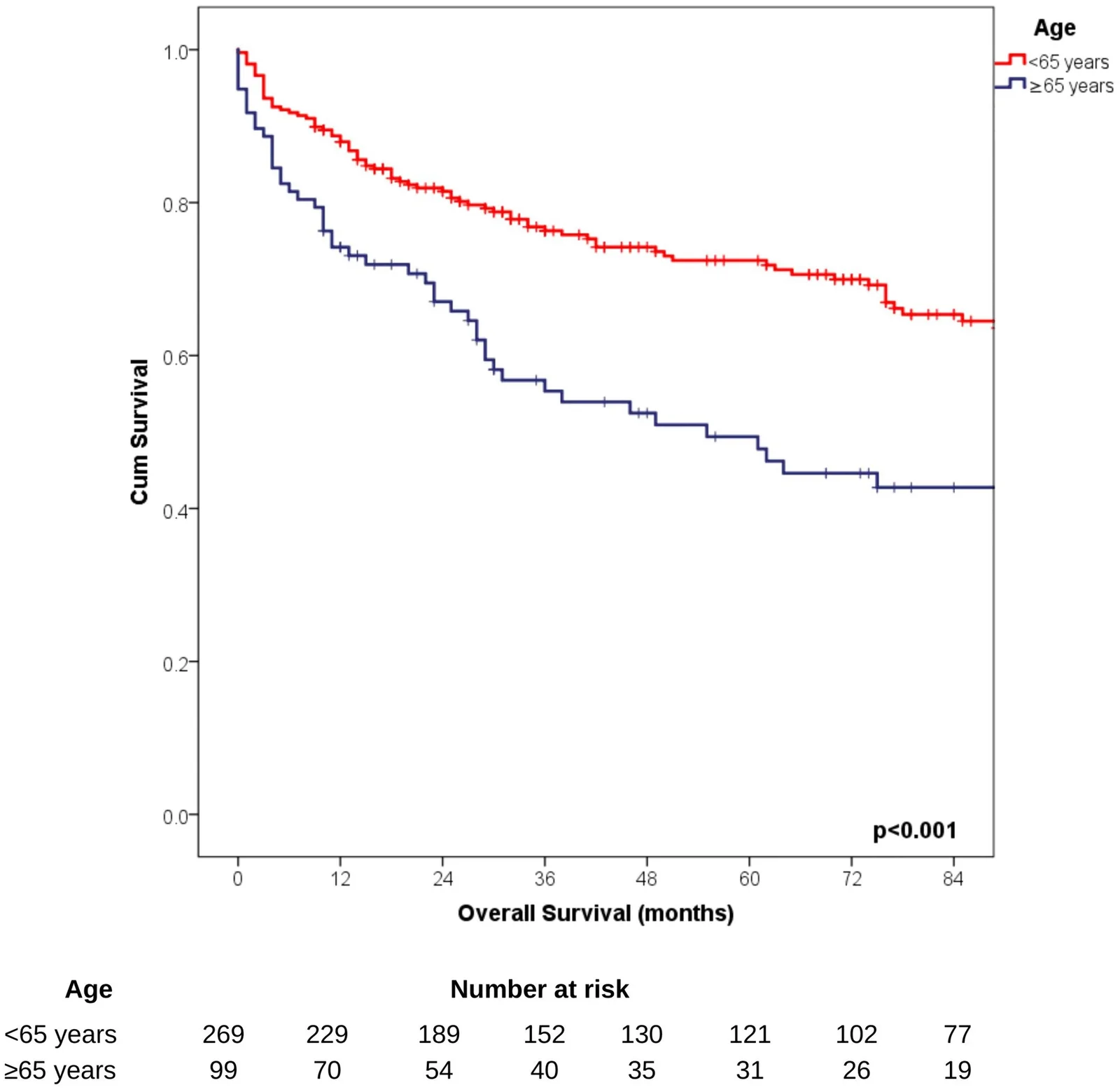
Kaplan-Meier curves showing overall survival stratified by age. Vertical marks indicate censored observations, and numbers at risk are shown below the plot. Corresponding 5-year overall survival estimates with 95% confidence intervals are reported in Table 3.

**Fig 5:**
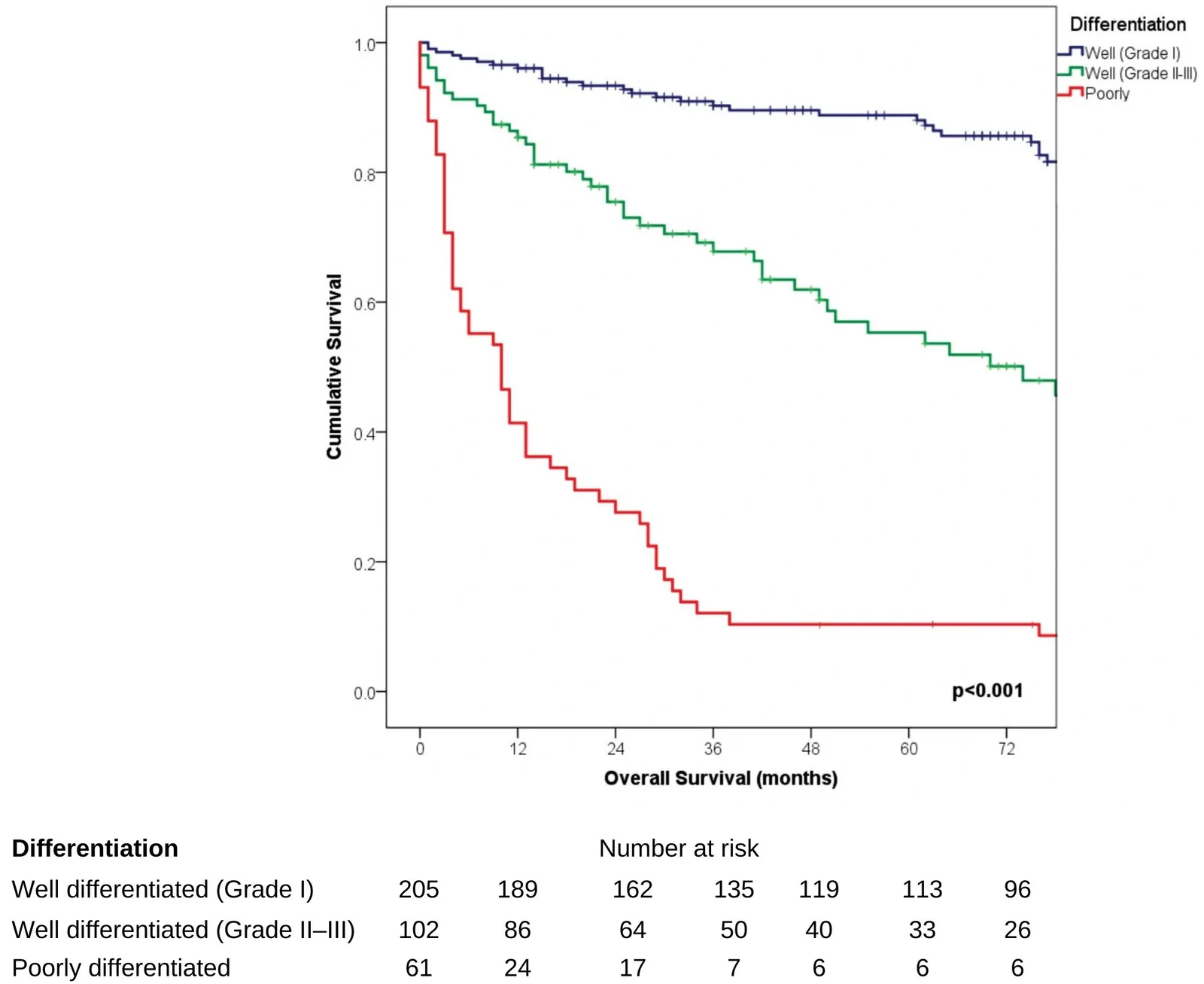
Kaplan-Meier curves showing overall survival according to tumor differentiation and grade. Vertical marks indicate censored observations, and numbers at risk are shown below the plot. Corresponding 5-year overall survival estimates with 95% confidence intervals are reported in Table 3.

Given the close relationship between tumor differentiation and grade, sensitivity analyses were performed using separate multivariable Cox models (S2 File, Supplementary Table 2). When tumor differentiation was included without grade (Model A), NEC histology remained independently associated with worse OS (aHR 5.76, 95% CI 3.59–9.23; p < 0.001). When tumor grade was included without differentiation (Model B), grade 2–3 disease was independently associated with worse OS (aHR 3.07, 95% CI 1.88–5.01; p < 0.001). Metastatic disease remained independently associated with worse OS in both models (Model A: aHR 2.96, 95% CI 1.87–4.70; p < 0.001; Model B: aHR 2.47, 95% CI 1.51–4.03; p < 0.001), and surgery remained associated with improved OS in both models (Model A: aHR 0.22, 95% CI: 0.14–0.37; p < 0.001; Model B: aHR 0.30, 95% CI: 0.18–0.49; p < 0.001). The proportional hazards assumption showed no evidence of global violation in either sensitivity model (Model A, p = 0.45; Model B, p = 0.33). The final multivariable Cox model included 13 estimated regression parameters and 140 observed deaths, corresponding to approximately 10 outcome events per regression parameter, consistent with conventional recommendations for Cox proportional hazards regression models.

Subgroup analyses according to disease extent are presented in S2 File (Supplementary Tables 3 and 4).. In the multivariable analyses, older age, NEC histology, and tumor grade 2–3 were independently associated with worse OS among patients with non-metastatic disease, whereas surgery was associated with improved OS. Among patients with metastatic disease, lower gastrointestinal tract and gallbladder primary tumors, as well as NEC histology, were independently associated with worse OS, whereas surgery and systemic treatment were associated with improved OS.

## Discussion

To the best of our knowledge, this retrospective study represents the largest single-center, hospital-based cohort of patients with GEP-NENs reported from Peru. It covers a 15-year period and includes 368 patients treated at a national referral cancer center.

According to primary tumor site, the majority of patients in our cohort presented with rNENs, and gNENs accounted for 39.1% and 19.8% of cases, respectively. In comparison, siNENs accounted for only 11.1%. The predominance of rNENs has been reported in other LATAM countries, such as Panama and Ecuador [15,16], as well as in Asian population-based registries, including those from China, Taiwan, and Japan [4–6]. Conversely, registries from neighboring countries, such as Chile and Argentina, report higher proportions of siNENs at 46% and 26.9%, respectively [10,17], consistent with population-based registries in the U.S., England, and Australia, which report siNENs as the predominant primary tumor site [1,2,7,8]. We hypothesize that differences in genetic ancestry may contribute to the development of GEP-NENs and their anatomic distribution; however, these findings should be interpreted cautiously because differences in diagnostic practices and healthcare infrastructure are also likely to influence the observed distribution across populations. Countries such as Peru, Ecuador, and Panama exhibit a predominant Native American ancestry, whereas Chile and Argentina show greater predominance of European ancestry [18,19]. This also aligns with the report by Paciorek et al [9], which demonstrated nearly two-fold higher age-adjusted incidence of rNENs among American Indian/Alaska Native individuals compared with non-Hispanic White individuals (IRR, 2.14; 95% CI, 1.23–3.51; p < 0.0001).

Nevertheless, comparisons of primary site distribution across LATAM cohorts should be interpreted with caution, as these differences may partly reflect diagnostic bias due to heterogeneity in diagnostic capacity, including differences in access to gastrointestinal endoscopy, functional imaging (particularly 68Ga-DOTATATE PET/CT), and specialized referral centers. Another plausible explanation is the rise in incidental detection of rNENs and gNENs in recent years through colorectal screening colonoscopy and upper gastrointestinal endoscopy, as reported in studies from England and Japan [20,21]. In Peru, the implementation of Plan Esperanza in 2012, the National Plan for Comprehensive Cancer Care and Improved Access to Oncologic Services, was associated with expanded access to diagnostic procedures, particularly gastrointestinal endoscopy [22,23]. Taken together, the available evidence suggests that GEP-NENs across Latin America share several common clinicopathological characteristics, including a predominance of well-differentiated tumors and relatively young age at diagnosis, whereas the distribution of primary tumor sites varies substantially among countries [20–23]. These differences are likely multifactorial and may reflect variation in ancestry, healthcare systems, referral patterns, and diagnostic capacity rather than true biological differences alone.

The median age at diagnosis was 57 years (IQR, 47–65), with 73.1% of patients younger than 65 years. This is concordant with other LATAM series, which generally report younger patient populations [10,17,24,25]. Similarly, a population-based registry from Beijing reports a median age of 56 years for extrapulmonary NETs [4], and a U.S. registry reports a median age of 58 years for GEP-NENs [1]. In our study, age at diagnosis varied by tumor differentiation and grade. The majority of GEP-NETs were younger than 65 years (76.8%), whereas nearly half of GEP-NECs were aged 65 years or older (46.6%). This finding aligns with data from Chinese and Japanese registries, which reported a higher prevalence of NECs among older patients [4,6]. The concordance of our findings with large population-based studies suggests that differences in age at onset are likely attributable to intrinsic tumor biology rather than underdiagnosis or limited healthcare access.

Regarding the distribution by sex, our cohort exhibited a female predominance of 56.2%. This correlates with numerous other reports [1,2,15,24,25]. In contrast, a male predominance has been documented in Asian population-based registries, including those from China, Japan, and Taiwan [3–6], as well as in other LATAM cohorts [10,16,17].

Based on tumor differentiation, we found that 84.2% of patients had NETs, whereas NECs accounted for only 15.8%. Moreover, there were significant differences by tumor differentiation and primary tumor site. All aNENs and approximately 90% of rNENs and siNENs were NETs, whereas all gbNENs were NECs. Similarly, in a U.S. population-based registry, among 44,733 patients, 81.9% were reported as NETs, whereas 18.1% were NECs [2]. Data from the Japanese registry indicated that at least 90% of siNENs, rNENs, and aNENs were NETs, with approximately 80% classified as G1 tumors. On the other hand, NENs from the upper digestive system were predominantly NECs [6]. Hispanic/Latino cohorts have also reported similar distributions: two Colombian registries reported a predominance of NETs at 93.6% and 95.6%, respectively [24,25], and the Argentine registry reported 89% of NETs among 441 GEP-NENs [10].

Surgery was the most frequently reported treatment modality, accounting for 68.2% of cases. In patients with G1 and G2 tumors, surgery represents the standard treatment approach with curative intent for localized disease [26]. In contrast, despite upfront surgical management, NEC patients experience high recurrence rates and have a poor prognosis [27]. In selected patients with metastatic disease, cytoreductive surgery removing at least 70% of the tumor burden has been associated with improved survival [28]. In our cohort, surgery remained independently associated with longer OS (aHR = 0.25, 95% CI: 0.15–0.41; p < 0.001). Similarly, systemic treatment was associated with improved OS (aHR = 0.53, 95% CI: 0.31–0.92; p = 0.024). Improved survival with chemotherapy has previously been reported in non-pNETs [29]. Furthermore, the phase III ECOG-ACRIN E2211 trial, which compared Capecitabine plus Temozolamide (CAPTEM) with temozolomide monotherapy in advanced pNETs, demonstrated a significant benefit for the combination regimen (HR, 0.58; p = 0.022) [30]. In contrast, despite remaining the standard first-line regimen for GEP-NECs, platinum-etoposide is associated with limited efficacy and dismal survival outcomes [31]. These findings remained consistent after stage-stratified analyses; however, residual confounding by indication cannot be excluded. Given the retrospective design, these treatment-related associations should be considered exploratory and not interpreted as evidence of causal treatment benefit.

The median OS for our study was 62 months, with a 5-year OS rate of 52%. Survival outcomes differed according to primary tumor site, sex, age, tumor grade, and differentiation in the univariate analyses. The most favorable outcomes were observed in aNENs, followed by siNENs, with 5-year OS rates of 83% and 64%, respectively. These findings are consistent with population-based studies from the U.S. and England, in which aNENs also demonstrated the longest 5-year overall survival among GEP-NENs [2,7]. In contrast, the poorest survival outcomes were observed in gbNENs, with an estimated 5-year OS of 18%. Notably, all cases were NECs, and the prognosis of gbNECs remains poor regardless of treatment modality, with a reported median OS of 4–6 months [31]. Interestingly, subgroup analyses according to disease extent suggested that the prognostic impact of primary tumor site was more evident among patients with metastatic disease, whereas no independent association was observed in the non-metastatic subgroup.

Five-year OS was significantly longer in females than in male patients, with 60% and 42% rates, respectively (p = 0.001). In univariate analysis, male sex was associated with a 73% higher risk of death (HR = 1.73; 95% CI, 1.24–2.42). These findings align with previous reports demonstrating favorable OS among female patients in unadjusted survival analyses, although sex was not independently associated with OS after multivariable adjustment in our cohort [3–5,7,10].. A population-based registry from Taiwan reported a 36% reduction in the risk of death among females compared with males in a multivariate analysis (HR = 0.64; 95% CI, 0.59–0.69) [5]. Similarly, a population-based registry from England identified male sex as an independent adverse prognostic factor, with a 43% increased risk of mortality (HR = 1.43; 95% CI, 1.35–1.51) [7]. The discrepancy between our findings and those of larger population-based registries may reflect differences in sample size, case mix, or residual confounding inherent to our single-center cohort. The reasons for this discrepancy remain unclear; however, it has been hypothesized that a higher expression of estrogen and progesterone receptors is associated with a favorable outcome in female patients [3].

Although older age was associated with worse overall survival in the univariate analysis, this association did not remain statistically significant after multivariable adjustment. In contrast, several population-based studies have identified older age as an independent adverse prognostic factor for survival [2,5,7,15,25]. The lack of an independent association in our cohort may reflect the relatively limited sample size and residual heterogeneity inherent to this single-center retrospective study.

By disease extent at diagnosis, non-metastatic disease was associated with a substantially favorable survival outcome, with a 5-year OS of 70% compared with 32% in metastatic disease. In the primary multivariable Cox model, metastatic disease was independently associated with worse OS (aHR = 2.61, 95% CI: 1.63–4.20; p < 0.001). This association remained robust in the sensitivity analyses in which tumor differentiation and grade were evaluated separately. Similarly, data from the U.S. registry showed that metastatic disease was associated with nearly a three-fold higher risk of death compared with localized disease (aHR = 2.96; 95% CI, 2.90–3.02) [2]. In addition, the England population-based study showed a two-fold risk of death in Stage IV disease compared to Stage I (HR = 2.11; 95% CI, 2.01–2.23) [7].

Tumor differentiation was strongly associated with OS. In the primary multivariable model, NEC histology was associated with worse OS compared with NET histology (aHR = 4.36, 95% CI: 2.59–7.31; p < 0.001). This association remained strong when tumor differentiation was evaluated without tumor grade in the sensitivity analysis (aHR = 5.76, 95% CI: 3.59–9.23; p < 0.001). Similarly, when grade was evaluated without differentiation, grade 2–3 disease was associated with worse OS compared with G1 disease (aHR = 3.07, 95% CI: 1.88–5.01; p < 0.001). In U.S. population-based data, NECs were linked to about a fourfold increased risk of death, whereas data from England indicated a 29% higher risk compared to NETs [2,7].

This study has several limitations that should be considered when interpreting the results. First, comprehensive AJCC/TNM staging data were not consistently recorded throughout the 15-year study period. Therefore, we categorized disease extent at diagnosis as non-metastatic (Stages I-III) or metastatic (Stage IV). Although this approach enabled standardized survival analyses across the cohort, it precluded evaluation of survival differences among individual AJCC/TNM stages and between localized and regional disease within the non-metastatic group. Second, treatment data for a subgroup of patients (n = 88) were incomplete, with inconsistent documentation of treatment intent, surgical radicality, treatment sequence, cytoreductive thresholds, and subsequent therapies. Therefore, treatment-related survival analyses should be interpreted with caution. Although additional stage-stratified analyses were performed, residual confounding by indication cannot be excluded. Third, cases lacking a complete pathological assessment and those categorized as metastatic disease of unknown primary were excluded, potentially leading to underrepresentation of specific tumor subgroups. This may have particularly affected siNENs, which are often difficult to identify due to their anatomical location and the frequent need for advanced functional imaging. Finally, because this was a single-center, hospital-based cohort from a national referral cancer center, the study was not designed to estimate national incidence or population-level survival outcomes in Peru. Referral bias may also have influenced the observed clinical profile, as patients referred to tertiary oncology centers are more likely to have complex disease, advanced presentation, or require specialized diagnostic and therapeutic approaches.

Emerging evidence suggests that the gut microbiota may contribute to the development, progression, and biological heterogeneity of GEP-NENs [32,33]. More broadly, emerging preclinical research has explored food–medicine homology and other complementary approaches in cancer, including bioactive food-derived compounds and deuterium-depleted water. Although these approaches remain investigational and their clinical relevance to GEP-NENs is unknown, they illustrate growing interest in metabolic and diet-related factors in cancer biology [34–36]. Our retrospective clinical cohort did not include microbiome, metagenomic, metabolomic, dietary, or environmental data, precluding evaluation of these potential mechanisms. Future prospective studies integrating clinical data with microbiome and metabolomic profiling may help clarify their relevance to GEP-NEN heterogeneity.

## Conclusion

This 15-year national referral-center cohort provides one of the largest descriptions of the clinicopathologic characteristics and survival of patients with GEP-NENs reported from Peru. Most tumors were well-differentiated NETs, while metastatic disease was associated with worse overall survival; higher grade and poor differentiation were also associated with worse outcomes when evaluated separately. Although these findings are not population-based and should not be generalized to all patients in Peru, the inclusion of patients referred from different geographic regions provides clinically relevant data from an underrepresented Latin American setting. Future national and regional registries, standardized data collection, and multicenter studies are needed to better characterize GEP-NENs and validate these findings across Latin America.

## Supporting information

S1 File Deidentified Excel dataset containing clinical and pathologic information on Peruvian patients diagnosed with Gastroenteropancreatic Neuroendocrine Neoplasms. (XLSX)

S2 File Supplementary Tables 1–4. Supplementary tables presenting treatment modalities according to disease extent at diagnosis, sensitivity multivariable Cox proportional hazards regression analyses, and univariate and multivariable Cox proportional hazards regression analyses stratified by non-metastatic and metastatic disease.
(DOCX)
