# Supplementary material for: Clinicopathologic characteristics and survival outcomes of gastroenteropancreatic neuroendocrine neoplasms in Peru: A 15-year retrospective cohort study
Source: PLoS One. 2026 Nov 5;21(11):e0361867. doi: 10.1371/journal.pone.0361867 (PMC13739108; doi:10.1371/journal.pone.0361867)
Supplement: Supplementary file 1 [file pone.0361867.s002.docx]

**Supplementary Table 1.** Treatment modalities according to disease extent at diagnosis in patients with gastroenteropancreatic neuroendocrine neoplasms.

| **Treatment modality** | **Non-metastatic (%)** | **Metastatic (%)** |
| --- | --- | --- |
| Palliative | 6 (3.7) | 32 (27.6) |
| Combined | 3 (1.8) | 2 (1.7) |
| Radiotherapy | 4 (2.5) | 3 (2.6) |
| Surgery | 143 (87.7) | 47 (40.5) |
| Systemic | 7 (4.3) | 32 (27.6) |
| Missing treatment data | 53 | 2 |

Data are presented as n (%). Percentages for treatment modalities were calculated among patients with available treatment information within each disease-extent group (non-metastatic, n=163; metastatic, n=116); missing treatment observations were excluded from percentage calculations and are reported separately.

**Supplementary Table 2.** Sensitivity multivariable Cox proportional hazards analyses of factors associated with overall survival, with tumor differentiation and tumor grade evaluated in separate models.

| **Variable** | **Model A: Tumor differentiation** | | | **Model B: Tumor grade** | | |
| --- | --- | --- | --- | --- | --- | --- |
|  | aHR | 95% CI | p-value | aHR | 95% CI | p-value |
| **Age group** |  |  |  |  |  |  |
| <65 | 1.00 | - | - | 1.00 |  |  |
| ≥65 | 1.37 | 0.91-2.06 | 0.133 | 1.78 | 1.21-2.64 | 0.004 |
| **Sex** |  |  |  |  |  |  |
| Female | 1.00 | - | - | 1.00 |  |  |
| Male | 0.64 | 0.42-0.97 | 0.036 | 0.69 | 0.46-1.03 | 0.072 |
| **ECOG** |  |  |  |  |  |  |
| ≤1 | 1.00 | - | - | 1.00 |  |  |
| 2 | 2.00 | 0.90-4.47 | 0.091 | 2.90 | 1.31-6.40 | 0.008 |
| **Primary tumor site** |  |  |  |  |  |  |
| Pancreas | 1.00 | - | - | 1.00 |  |  |
| Lower gastrointestinal tract | 2.70 | 1.49-4.87 | 0.001 | 2.54 | 1.43-4.52 | 0.001 |
| Gallbladder | 2.52 | 1.49-4.87 | 0.014 | 4.90 | 2.00-11.99 | 0.001 |
| Small bowel | 1.87 | 0.76-4.60 | 0.170 | 3.24 | 1.55-6.78 | 0.002 |
| Stomach/esophagus | 2.16 | 1.08-4.33 | 0.030 | 3.23 | 1.60-6.50 | 0.001 |
| **Disease extent at diagnosis** |  |  |  |  |  |  |
| Non-metastatic | 1.00 | - | - | 1.00 |  |  |
| Metastatic | 2.96 | 1.87-4.70 | <0.001 | 2.47 | 1.51-4.03 | <0.001 |
| **Tumor differentiation** |  |  |  |  |  |  |
| NET | 1.00 | - | - | - |  |  |
| NEC | 5.76 | 3.59-9.23 | <0.001 | - | - | - |
| **Treatment modalities** |  |  |  |  |  |  |
| Palliative | 1.00 | - | - | 1.00 |  |  |
| Combined and radiotherapy | 0.50 | 0.20-1.27 | 0.145 | 1.05 | 0.42-2.59 | 0.920 |
| Surgery | 0.22 | 0.14-0.37 | <0.001 | 0.30 | 0.18-0.49 | <0.001 |
| Systemic | 0.52 | 0.30-0.91 | 0.023 | 0.59 | 0.35-1.02 | 0.058 |
| **Tumor grade** |  |  |  |  |  |  |
| G1 | - | - | - | 1.00 |  |  |
| G2-3 | - | - | - | 3.07 | 1.88-5.01 | <0.001 |

Model A included tumor differentiation and excluded tumor grade. Model B included tumor grade and excluded tumor differentiation.

The proportional hazards assumption was assessed using Schoenfeld residuals, with no evidence of global violation in either model (Model A, p=0.45; Model B, p=0.33).

**Abbreviations:** aHR, adjusted hazard ratio; CI, confidence interval; ECOG, Eastern Cooperative Oncology Group; NEC, poorly differentiated neuroendocrine carcinoma; NET, well-differentiated neuroendocrine tumor.

**Supplementary Table 3.** Univariate and multivariate Cox proportional hazards analysis of factors associated with overall survival in patients with non-metastatic gastroenteropancreatic neuroendocrine neoplasms.

| **Variable** | **HR** **(95% CI)** | **p-value** | **aHR (95% CI)** | **p-value** |
| --- | --- | --- | --- | --- |
| **Age (years)** | 1.04 (1.02-1.06) | 0.001 | 1.03 (1.01-1.07) | 0.026 |
| **Sex** |  |  |  |  |
| Female | 1.00 | - | 1.00 | - |
| Male | 0.35 (0.20-0.63) | <0.001 | 0.32 (0.15-0.66) | 0.002 |
| **ECOG** |  |  |  |  |
| ≤1 | 1.00 | - | 1.00 | - |
| 2 | 6.45 (2.70-17.55) | <0.001 | 1.31 (0.82-2.49) | 0.359 |
| **Primary tumor site** |  |  |  |  |
| Pancreas | 1.00 | - | 1.00 | - |
| Lower gastrointestinal tract | 0.69 (0.41-2.06) | 0.891 | 0.62 (0.19-2.03) | 0.428 |
| Gallbladder | 0.87 (0.36-2.08) | 0.754 | 0.55 (0.24-2.14) | 0.551 |
| Small bowel | 0.90 (0.52-1.60) | 0.193 | 0.77 (0.21-2.81) | 0.692 |
| Stomach/esophagus | 5.93 (1.22-10.75) | 0.027 | 2.00 (0.32-9.51) | 0.458 |
| **Tumor differentiation** |  |  |  |  |
| NET | 1.00 | - | 1.00 | - |
| NEC | 9.87 (5.37-18.14) | <0.001 | 4.86 (1.79-9.67) | 0.002 |
| **Treatment modalities** |  |  |  |  |
| Palliative, combined, radiotherapy and systemic | 1.00 | - | 1.00 | - |
| Surgery | 0.21 (0.10-0.41) | <0.001 | 0.27 (0.11-0.64) | 0.003 |
| **Tumor grade** |  |  |  |  |
| G1 | 1.00 | - | 1.00 | - |
| G2-3 | 6.39 (3.55-11.49) | <0.001 | 2.90 (1.21-6.96) | 0.017 |

**Abbreviations:** aHR, adjusted hazard ratio; CI, confidence interval; ECOG, Eastern Cooperative Oncology Group; HR, hazard ratio; NEC, poorly differentiated neuroendocrine carcinoma; NET, well-differentiated neuroendocrine tumor.

**Supplementary Table 4.** Univariate and multivariate Cox proportional hazards analysis of factors associated with overall survival in patients with metastatic gastroenteropancreatic neuroendocrine neoplasms.

| **Variable** | **HR** **(95% CI)** | **p-value** | **aHR (95% CI)** | **p-value** |
| --- | --- | --- | --- | --- |
| **Age (years)** | 1.02 (1.01-1.04) | 0.007 | 1.01 (0.98-1.03) | 0.484 |
| **Sex** |  |  |  |  |
| Female | 1.00 | - | 1.00 | - |
| Male | 1.11 (0.72-1.72) | 0.631 | 1.09 (0.64-1.86) | 0.761 |
| **ECOG** |  |  |  |  |
| ≤1 | 1.00 | - | 1.00 | - |
| 2 | 4.77 (1.97-11.55) | 0.001 | 2.48 (0.90-6.89) | 0.081 |
| **Primary tumor site** |  |  |  |  |
| Pancreas | 1.00 | - | 1.00 | - |
| Lower gastrointestinal tract | 4.44 (1.98-9.95) | <0.001 | 3.85 (1.44-9.26) | 0.007 |
| Gallbladder | 3.04 (1.62-5.70) | 0.001 | 4.47 (2.13-9.38) | <0.001 |
| Small bowel | 0.98 (0.40-2.42) | 0.972 | 1.52 (0.47-4.91) | 0.481 |
| Stomach/esophagus | 4.39 (1.74-11.07) | 0.002 | 1.77 (0.60-5.24) | 0.306 |
| **Tumor differentiation** |  |  |  |  |
| NET | 1.00 | - | 1.00 | - |
| NEC | 3.10 (1.97-4.89) | <0.001 | 5.21 (2.65-9.26) | <0.001 |
| **Treatment modalities** |  |  |  |  |
| Palliative | 1.00 | - | 1.00 | - |
| Combined and radiotherapy | 0.36 (0.18-1.64) | 0.615 | 0.45 (0.12-1.68) | 0.233 |
| Surgery | 0.28 (0.17-0.49) | <0.001 | 0.21 (0.11-0.38) | <0.001 |
| Systemic | 0.61 (0.35-0.98) | 0.032 | 0.37 (0.20-0.68) | 0.001 |
| **Tumor grade** |  |  |  |  |
| G1 | 1.00 | - | 1.00 | - |
| G2-3 | 1.50 (0.86-2.61) | 0.150 | 0.85 (0.41-1.77) | 0.662 |

**Abbreviations:** aHR, adjusted hazard ratio; CI, confidence interval; ECOG, Eastern Cooperative Oncology Group; HR, hazard ratio; NEC, poorly differentiated neuroendocrine carcinoma; NET, well-differentiated neuroendocrine tumor.
